# Cover of Le Vie d'Italia magazine from 1924

**DOI:** 10.3201/eid0706.000000

**Published:** 2001

**Authors:** Roberto Romi

**Affiliations:** * Instituto Superiore di Sanità, Rome, Italy


					News and Notes
Vol. 7, No.6, November-December 2001 1073 Emerging Infectious Diseases
Cover of Le Vie d'Italia magazine from 1924
Provided courtesy of Dr. Guido Sabatinelli.
Le Vie d'Italia was established near the end of the 19th
century in Milan as the monthly magazine of the Touring
Club of Italy. Some 30 years later, in the 1920s, the magazine
took on the additional role of Bulletin of the (then informal)
National Organization for Tourism in Rome. Today, the mag-
azine, which has been very popular, is still published by the
Touring Club of Italy under the name Qui Touring.
The issue of Le Vie d'Italia featured on this cover of
Emerging Infectious Diseases is from August 1924 (Year
XXX, No.8, circulation 150,000). The Italian text reads,
"Monthly Magazine of the Touring Club of Italy, Milano, 10
Corso Italia-Roads of Italy, Official Bulletin of the National
Body of Tourist Enterprises."
In the center of this Le Vie d'Italia cover, under the
image of the mosquito looming over the water, the inscrip-
tion reads, "From the painting by E. Serra, Evening in the
Pontine Marshes." The Pontine marshes, 50 Km south of
Rome, were at that time the most malarious area of conti-
nental Italy-today the area is one of the most fertile plains
in the country.
In the lower part of the cover, the Italian text reads,
"Esanofele tablets, Esanofelina syrup for children, against
malaria fever." Esanofele was produced by the F. Bisleri
Company, which also produced a famous liqueur, Ferrochina
Bisleri, probably an alcohol infusion of cinchona bark, herbs,
and iron salts. In 1924, quinine was produced only under a
state monopoly, so Esanofele was probably also an herbal
preparation with perhaps some antimalarial activity due to
the cinchona bark.
Roberto Romi
Laboratorio di Parassitologia
Istituto Superiore di Sanita
Rome, Italy
The Cover
Large-Scale Spread of an
Emerging Pathogen by Shipping
Other Articles Include
The Dioxin Crisis as Experiment To Determine
Poultry-Related Campylobacter Enteritis
A Large Outbreak of Legionnaires' Disease
at a Flower Show, the Netherlands, 1999
Tularemia Outbreak Investigation in Kosovo:
Case-Control and Environmental Studies
African Trypanosomiasis in Travelers
Returning to the United Kingdom
Absence of Mycoplasma Contamination
in the AVA Anthrax Vaccine
For a complete list of articles included in
the January-February issue, and for articles
published online ahead of print publication, see
http://www.cdc.gov/ncidod/eid/upcoming.htm
In the next issue of

				

**Figure:**
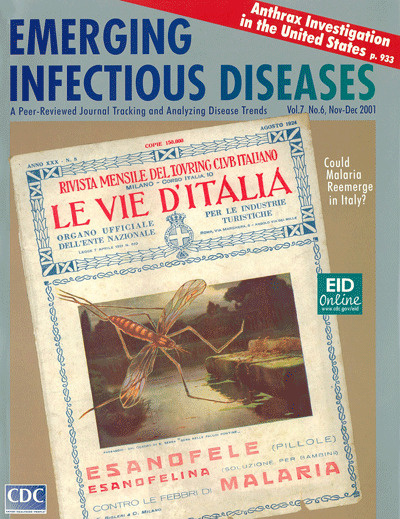
Cover of Le Vie d’Italia magazine from 1924 provided courtesy of Dr. Guido Sabatinelli

Le Vie d'Italia was established near the end of the 19th century in Milan as the monthly magazine of the Touring Club of Italy. Some 30 years later, in the 1920s, the magazine took on the additional role of Bulletin of the (then informal) National Organization for Tourism in Rome. Today, the magazine, which has been very popular, is still published by the Touring Club of Italy under the name Quì Touring.

The issue of Le Vie d'Italia featured on this cover of Emerging Infectious Diseases is from August 1924 (Year XXX, No.8, circulation 150,000). The Italian text reads, "Monthly Magazine of the Touring Club of Italy, Milano, 10 Corso Italia–Roads of Italy, Official Bulletin of the National Body of Tourist Enterprises."

In the center of this Le Vie d'Italia cover, under the image of the mosquito looming over the water, the inscription reads, "From the painting by E. Serra, Evening in the Pontine Marshes."The Pontine marshes, 50 Km south of Rome, were at that time the most malarious area of continental Italy–today the area is one of the most fertile plains in the country.

In the lower part of the cover, the Italian text reads, "Esanofele tablets, Esanofelina syrup for children, against malaria fever."Esanofele was produced by the F. Bisleri Company, which also produced a famous liqueur, Ferrochina Bisleri, probably an alcohol infusion of cinchona bark, herbs, and iron salts. In 1924, quinine was produced only under a state monopoly, so Esanofele was probably also an herbal preparation with perhaps some antimalarial activity due to the cinchona bark.

